# Exogenous Alanine Reverses the Bacterial Resistance to Zhongshengmycin with the Promotion of the P Cycle in *Xanthomonas oryzae*

**DOI:** 10.3390/antibiotics11020245

**Published:** 2022-02-14

**Authors:** Yi Guan, Peihua Shen, Meiyun Lin, Xiuyun Ye

**Affiliations:** Fujian Key Laboratory of Marine Enzyme Engineering, College of Biological Science and Engineering, Fuzhou University, Fuzhou 350116, China; 200827045@fzu.edu.cn (P.S.); 200827031@fzu.edu.cn (M.L.)

**Keywords:** *Xanthomonas oryzae*, antibiotic resistance, Zhongshengmycin, P cycle, alanine

## Abstract

Microbial antibiotic resistance has become a worldwide concern, as it weakens the efficiency of the control of pathogenic microbes in both the fields of medicine and plant protection. A better understanding of antibiotic resistance mechanisms is helpful for the development of efficient approaches to settle this issue. In the present study, GC-MS-based metabolomic analysis was applied to explore the mechanisms of Zhongshengmycin (ZSM) resistance in *Xanthomonas oryzae* (Xoo), a bacterium that causes serious disease in rice. Our results show that the decline in the pyruvate cycle (the P cycle) was a feature for ZSM resistance in the metabolome of ZSM-resistant strain (Xoo-ZSM), which was further demonstrated as the expression level of genes involved in the P cycle and two enzyme activities were reduced. On the other hand, alanine was considered a crucial metabolite as it was significantly decreased in Xoo-ZSM. Exogenous alanine promoted the P cycle and enhanced the ZSM-mediated killing efficiency in Xoo-ZSM. Our study highlights that the depressed P cycle is a feature in Xoo-ZSM for the first time. Additionally, exogenous alanine is a candidate enhancer and can be applied with ZSM to improve the antibiotic-mediated killing efficiency in the control of infection caused by Xoo.

## 1. Introduction

*Xanthomonas oryzae* pv. oryzicola (Xoo) is a Gram-negative bacterium that infects rice and causes bacterial blight (BB), also called bacterial leaf blight (BLB). Foliar symptoms of BB usually become evident at the tillering stage as small, green water-soaked spots at the tips and margins of fully developed leaves. The spots expand along the veins, merge, and become chlorotic and then necrotic, forming opaque, white to grey-colored lesions that typically extend from the leaf tip down along the leaf veins and margins [1,2]. BB/BLB is one of the most serious bacterial diseases of rice and may result in a large loss in crop yields [3,4]. To protect crops from BB disease, various antibiotics, such as aristeromycin, formycin, shenqinmycin, and streptomycin, are applied for the control of *Xanthomonas oryzae* [5,6,7,8].

Even though the antibiotics show high efficiency in pathogen control, the overuse and long-term usage of antibiotics arouse a new widespread problem, microbial antibiotic resistance. Xoo with antibiotic resistance has been discovered, which poses a threat to the prevention and control of the BB disease [9,10]. Developing new antibiotics or breeding antibacterial varieties are solutions for solving this problem, but both are time consuming and labor intensive. Therefore, it is imperative to explore more approaches to settle the antibiotic resistance issue in Xoo control.

Zhongshengmycin (ZSM herein) is a kind of aminoglycoside antibiotic developed by the Chinese Academy of Agricultural Sciences and has been proved to prevent the plant diseases caused by Xoo with high efficiency [11]. In addition to its function in Xoo control, ZSM also has been proven to inhibit the hyphal growth in *Didymella segeticola*, a fungal phytopathogen that causes tea leaf spot disease [12], and show antimicrobial activity against several pathogens of kiwifruit [13]. However, ZSM-resistant bacteria have also been isolated in recent years [14]. Thus, it is urgent to unveil the mechanism of bacterial ZSM resistance so that novel strategies can be proposed to deal with antibiotic resistance for crop protection.

A line of evidence has shown that the different metabolic state of the microbes in vivo determines their sensitivity/resistance to antibiotics. For example, the enhanced biosynthesis of fatty acids is associated with the acquisition of ciprofloxacin resistance in *Edwardsiella tarda* [15]. In *Vibrio alginolyticus*, the depressed central carbon and energy metabolisms are associated with the acquisition of levofloxacin resistance [16]. The pyruvate cycle (P cycle) is a recently defined metabolic pathway that is considered as a merging of the PEP–pyruvate–AcCoA pathwayand the TCA cycle, and operates routinely as a general mechanism for energy production and regulation. The P cycle has been proven to be associated with bacterial aminoglycoside antibiotic resistance in *Escherichia coli* and *Edwardsiella tarda* [17]. Based on these findings, the microbial resistance to antibiotics could theoretically be reversed by metabolomic modulation, called metabolome reprogramming [18]. Therefore, exogenous metabolites or some enzyme inhibitors were introduced to turn the antibiotic-resistant metabolism into an antibiotic-sensitive metabolism in microbes and promote the efficiency of antibiotics. A recent study showed that the synergy of alanine and gentamicin reduces intracellular nitric oxide and elevates the gentamicin-mediated killing efficacy against gentamicin-resistant *Vibrio alginolyticus* [18]. On the other hand, reactive oxygen species (ROS)-related ceftazidime resistance caused by the P cycle is reverted by Fe^3+^ in *Edwardsiella tarda* [19]. Our previous work showed that the depression of fatty acid biosynthesis by triclosan reverses the ZSM resistance in Xoo [20]. Therefore, analysis of the metabolomic characterizations in vivo helps us to understand the mechanisms of antibiotic resistance and provides strategies to reverse bacterial antibiotic resistance.

In this study, GC-MS-based metabolomics were used to characterize the metabolomic features of Xoo-ZSM compared with those of Xoo-S. Xoo-S was sensitive to ZSM, while Xoo-ZSM was a derivative strain sequentially cultured in medium with 1/2 MIC, the MIC of which was 32-fold higher than that of Xoo-S. The depression of the P cycle was determined as a metabolic feature in Xoo-ZSM. Exogenous alanine promoted the P cycle, and further elevated the sensitivity of Xoo-ZSM to the ZSM. Our findings expand the understanding of metabolic features in antibiotic-resistant Xoo, and further propose a novel approach for the control of BB disease. The results are reported as follows.

## 2. Results

### 2.1. Characterization of the Antibiotic Resistance-Related Features of Xoo-S and Xoo-ZSM

**Figure 1:**
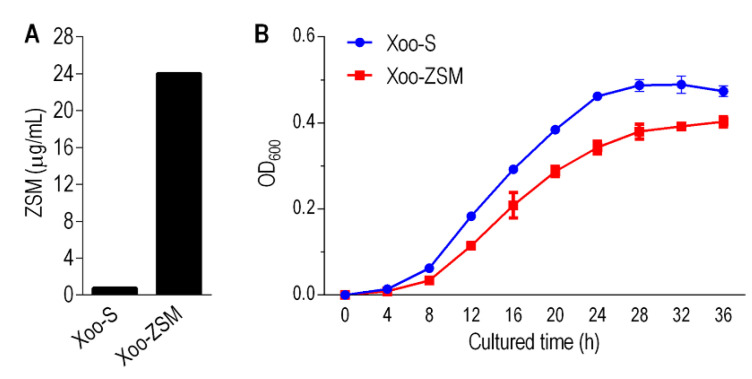
The features of strain Xoo-S and Xoo-ZSM. (**A**) The minimum inhibitory concentration (MIC) of Xoo-S and Xoo-ZSM with zhongshengmycin stress. (**B**) Growth curve of Xoo-S and Xoo-ZSM. Bars: SD calculated from three biological replicates.

Xoo-S refers to a Xanthomonas oryzae that is sensitive to zhongshengmycin (ZSM herein) with a minimum inhibitory concentration (MIC) of 0.75 μg/mL. Xoo-ZSM is a lab-generated zhongshengmycin-resistant strain with an MIC of 24 μg/mL, which was 32-fold higher than that of Xoo-S (Figure 1A). The growth curves of the two strains were assayed. The OD_600_ of the two strains cultured at 30 °C and 200 rpm were measured every 4 h from 0 h to 36 h. The results show that the growth trends have no significant differences between the two strains. The growth rate of both bacterial strains leveled off gradually after being cultured for 28 h. In addition, the OD_600_ of Xoo-ZSM was 19.84% lower than that of Xoo-S after being cultured for 32 h (Figure 1B), indicating the reduced biomass in the antibiotic-resistant strain compared with that in Xoo-S.

### 2.2. Profile of the Metabolomic Analysis

According to the growth curves of the two Xoo strains, 32 h-cultured products of Xoo-S and Xoo-ZSM were used for metabolomic analysis and subsequent experiments to explore the mechanism of zhongshengmycin resistance in *Xanthomonas oryzae*. After ultrasonication, vacuum rotary drying, methoxyamine hydrochloride co-incubation, and derivatization, the samples were used for the GC-MS test. Five biological individuals and two technical replicas for each strain were performed. In the present study, a total of 65 metabolites were obtained after removing the ribitol, which was added as the internal standard, and merging the same compounds. The correlation coefficients were higher than 0.995 between technical replicates, indicating a high reproducibility in the data collection phase (Figure S1A). All the identified metabolites can be categorized into carbohydrate (34%), amino acid (17%), lipid (34%), nucleotide (4%) and others (11%) (Figure S1B). The relative abundances are shown in the heat map (Figure S1C).

**Figure 2:**
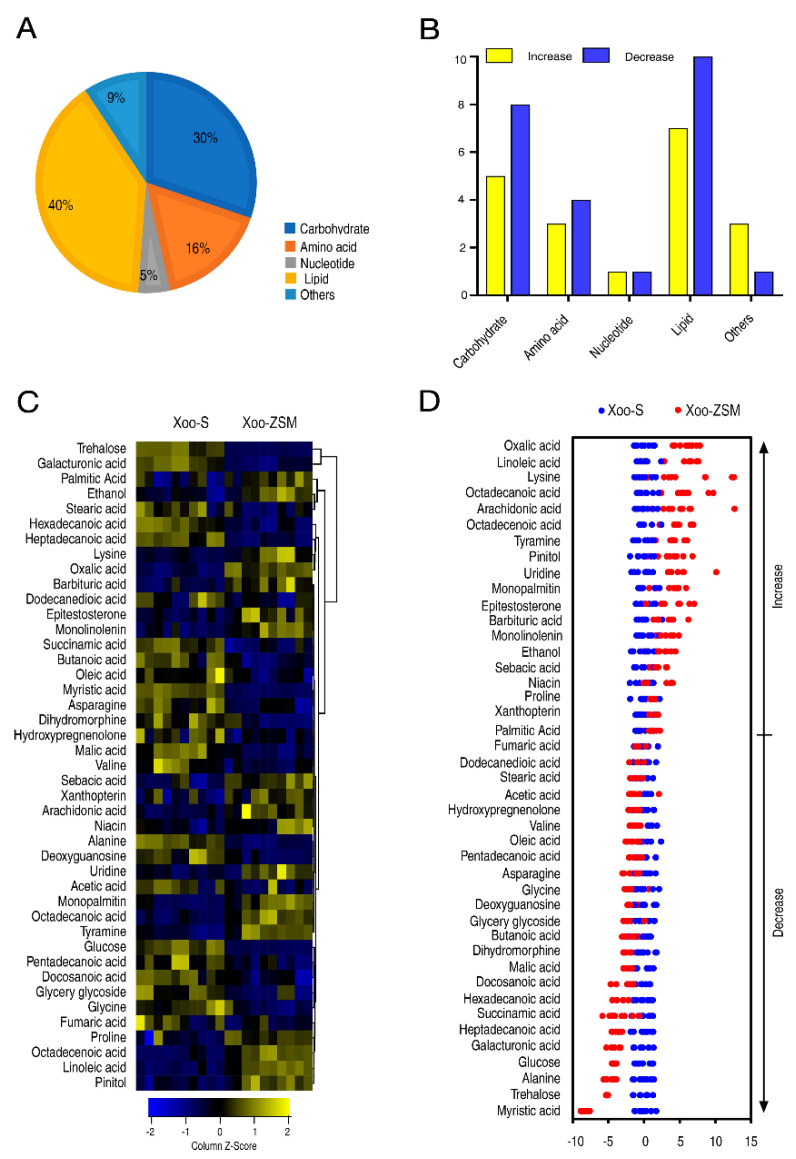
The differential metabolomic state between Xoo-S and Xoo-ZSM. (**A**) Percentages of differentially abundant metabolites in every category. (**B**) The numbers of increased and decreased DAMs in every category. The increase and decrease referred to the greater or lesser abundance of the DAMs in Xoo-ZSM when compared with that in Xoo-S. (**C**) Heat map of differentially abundant metabolites. Yellow and blue indicate an increase and decrease in the metabolites scaled to the mean and SD of the metabolite row, respectively (see color scale). Five biologic replicates and two technologic replicas in each group were performed, yielding a total of 20 datasets. (**D**) Z-score plots of differential abundances of metabolites based on control. The data from Xoo-ZSM are separately scaled to the mean and SD of Xoo-S. Each point represents one metabolite in one biological repeat and is colored by sample type.

Among the 65 metabolites, 43 were identified as differently abundant metabolites (DAMs) with a *p*-value < 0.05 according to the Mann–Whitney test. Among them, carbohydrate, amino acid, nucleotide, lipid, and others occupied 30%, 16%, 5%, 40%, and 9%, respectively (Figure 2A). As compared with Xoo-S, Xoo-ZSM exhibited different numbers of the increased or decreased DAMs according to each category (Figure 2B). The relative abundances were displayed in the heat map (Figure 2C). The Z values of the abundances of DAMs in Xoo-ZSM ranged from −10 to 15 compared with that in Xoo-S. The abundance of 19 and 24 metabolites was increased and decreased, respectively, in Xoo-ZSM, as compared with Xoo-S (Figure 2D).

In addition, orthogonal partial least-squares discriminant analysis (OPLS-DA) was applied for the recognition of the sample patterns, followed by ranking the altered abundance of metabolites in loading. The component (t[1]) separated Xoo-S from Xoo-ZSM (Figure S2A). The varied abundances of candidate metabolites were analyzed (Figure S2B). Combined with the Z-score and OPLS-DA results, we sorted glucose, ethanol, galacturonic acid, glycine, hexadecanoic acid, lysine, niacin, trehalose, and alanine as biomarkers which may influence the development of ZSM resistance in Xoo. Scatter diagrams of the biomarkers are displayed in Figure S3. These results indicate that the metabolomic situations were greatly different between Xoo-S and Xoo-ZSM.

### 2.3. Pathways Enrichment of the DAMs

**Figure 3:**
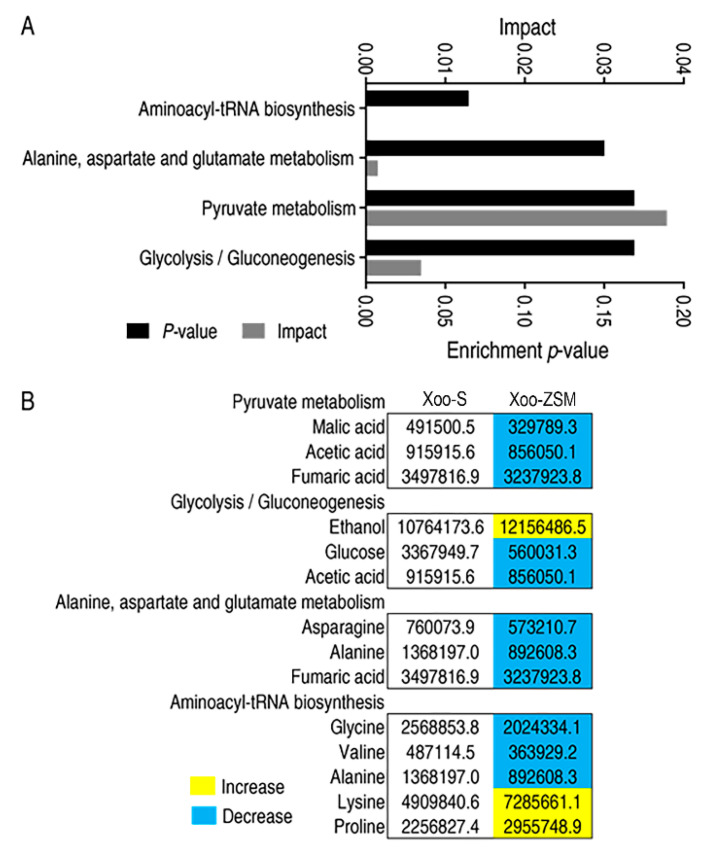
Metabolic variation in Xoo-ZSM. (**A**) Enrichment of significantly infected metabolic pathways in Xoo-ZSM. (**B**) Integrative analysis of metabolites in significantly enriched pathways. Yellow and blue indicate increased and decreased metabolites, respectively.

The DAMs were submitted to MetaboAnalyst (http://www.metaboanalyst.ca, accessed on 30 December 2021) and four pathways were significantly enriched, with a *p*-value < 0.05. The specific impact factor and *p*-value for each pathway are shown in Figure 3A. Among the four enriched pathways, alanine, aspartate and glutamate metabolism, pyruvate metabolism, and glycolysis/gluconeogenesis pathways were associated with metabolism, while the other one, aminoacyl-tRNA biosynthesis, was associated with genetic information processing. Detailed information about the abundance of each DAMs in Xoo-S or Xoo-ZSM is listed in Figure 3B. These findings suggest that metabolism may play a crucial role in the development of ZSM resistance in Xoo.

### 2.4. Reduced P Cycle in Xoo-ZSM

**Figure 4:**
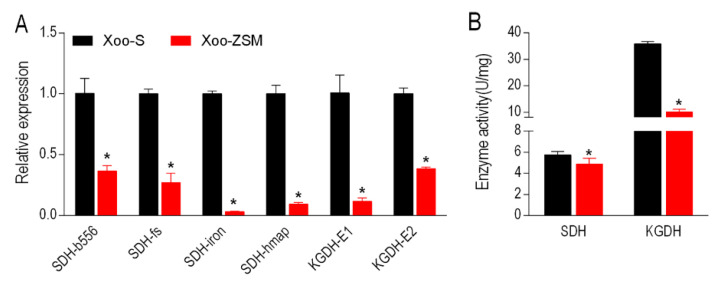
The attenuated P cycle in Xoo-ZSM. (**A**) The decreased expression levels of genes encoding two key enzymes in the P cycle. (**B**) The reduced activities of SDH and KGDH in Xoo-ZSM. The results are displayed as mean with SD, and significant differences were identified by Student’s *t*-test (* *p* < 0.05). At least three biological replicates were carried out.

Interestingly, we noticed that among the enriched pathways, alanine, aspartate and glutamate metabolism, pyruvate metabolism, and glycolysis/gluconeogenesis pathways were associated with the pyruvate cycle (the P cycle), a recently identified cycle providing respiratory energy in bacteria [17]. The significant decline in metabolites involved in these three pathways indicates a depression in the P cycle in Xoo-ZSM. To verify this hypothesis, the expression levels of genes associated with the P cycle were determined by qRT-PCR with specific primers (Table S1). The results show that four genes encoding succinate dehydrogenase (SDH), and two genes encoding α-ketoglutarate dehydrogenase (KGDH) were down-regulated in Xoo-ZSM (Figure 4A). We further tested the activities of the two enzymes in Xoo-S and Xoo-ZSM. Consistently, the activities of the two enzymes were decreased in Xoo-ZSM (Figure 4B). Therefore, the reduced expression levels of genes associated with the P cycle and the decreased SDH and KGDH activities suggests a depressed P cycle in Xoo-ZSM.

### 2.5. Elevated Sensitivity to ZSM and Upregulated P Cycle in the Presence of Exogenous Alanine

**Figure 5:**
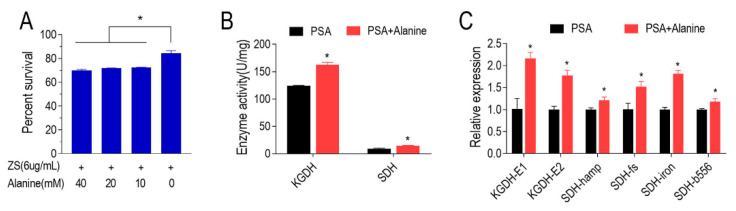
Exogenous alanine enhances the killing efficiency of ZSM and promotes the P cycle in ZSM-resistant Xoo. (**A**) The percent survival of ZSM-resistant Xoo in the PSA broth with gradient dosages of alanine from 0 to 40 mM. (**B**) Activities of KGDH and SDH in the ZSM-resistant Xoo in the presence or absence of 40 mM alanine. (**C**) The expression level of genes encoding KGDH and SDH in ZSM-resistant Xoo in the presence or absence of 40 mM alanine. The results are displayed as mean with SD, and significant differences are identified as determined by Student’s *t*-test (* *p* < 0.05). At least three biological replicates were carried out.

Alanine was one of the top three decreased metabolites in Xoo-ZSM, and the significantly reduced alanine, aspartate and glutamate metabolism pathway motivated us to investigate the influence of alanine on ZMS resistance in Xoo. The ZSM-resistant strain was cultured under the stress of 6 μg/mL ZSM and with gradient dosages of alanine ranging from 0 to 40 mM. A minimum of 10 mM alanine was required to achieve a ~12% reduction in percent survival, while any more did not seem to significantly further decrease the percent survival (Figure 5A), indicating that the exogenous alanine could enhance the efficiency of ZSM killing on Xoo. We further assayed the P cycle in the ZSM-resistant strain cultured with or without alanine. The enzyme activities of KGDH and SDH in ZSM-resistant Xoo cultured with alanine were both higher than those who cultured without alanine (Figure 5B). Furthermore, the results of qRT-PCR confirmed the elevation of the two enzyme activities in the P cycle (Figure 5C). Our results suggest that the alanine reverses the ZSM resistance and promotes the P cycle in the ZSM-resistant Xoo strain.

## 3. Discussion

In the present study, metabolomic characterizations of two Xoo strains with different sensitivities to zhongshengmycin (ZSM) were compared and analyzed. The results show that the decline in the P cycle was a feature in Xoo-ZSM. Moreover, exogenous alanine promoted the P cycle and reversed the antibiotic resistance in the ZSM-resistant strain. The detailed results are discussed as follows.

Firstly, Xoo-S and Xoo-ZSM were used for the metabolomic analysis and experimental performance. The growth curve of the two strains showed that Xoo-ZSM grew with a similar trend to Xoo-S, except for a relatively lower biomass, when compared with Xoo-S after the exponential phase. Interestingly, the tradeoff in growth seems to be a common phenotype in antibiotic-resistant strains, which may be attributed to the altered metabolism state, as shown in the central carbon metabolism, amino acid metabolism, and energy metabolism. For example, ciprofloxacin-resistant *Edwardsiella tarda* LTB4-R_CIP_ grew slower than ciprofloxacin-sensitive LTB4-S in the first 8 h, and showed a disrupted pyruvate cycle, decreased energy metabolism, and increased fatty acid biosynthesis [15]. The absence of *nqrA* or *nqrF* in *Vibrio alginolyticus* increased bacterial resistance to amikacin, gentamicin, and kanamycin, but led to reduced growth in the exponential phase. On the other hand, the metabolomics analysis revealed the promoted L-alanine catabolism and inhibited L-alanine anabolism [21]. As the metabolic condition may contribute to the bacterial growth state and the antibiotic resistance, the metabolomic data of the two strains were obtained by GC-MS, a widely used method for the research of bacterial antibiotic resistance [21,22,23]. Our work showed that the abundances of 65 metabolites varied in Xoo-ZSM compared with those in Xoo-S, of which 43 were identified as significantly differently abundant metabolites (DAMs) with a *p*-value < 0.05. In addition, Z-score analysis showed that 19 DAMs were increased while 24 DAMs were decreased in Xoo-ZSM when compared with that in Xoo-S. Furthermore, the 43 DAMs were submitted to MetaboAnalyst for KEGG pathway analysis. Four mainly infected pathways were enriched, while three of them, i.e., alanine, aspartate and glutamate metabolism, pyruvate metabolism, and glycolysis/gluconeogenesis, were associated with the metabolic category, and the other, aminoacyl-tRNA biosynthesis, was associated with genetic information processing, indicating that the variation in metabolism may play an important role in the ZSM-resistance development in Xoo. These results are consistent with previous reports that metabolic fluctuation affects the development of microbial antibiotic resistance [20,24,25,26].

Secondly, we noticed that among the KEGG-enriched metabolic pathways, alanine, aspartate and glutamate metabolism, pyruvate metabolism, and glycolysis/gluconeogenesis are associated with the pyruvate cycle (the P cycle), a novel metabolic pathway that was recently proposed to provide energy to bacteria and affect the antibiotic uptake [17,26]. According to previous studies, the P cycle usually showed a down-regulated pattern in various antibiotic-resistant bacteria. For example, a depressed P cycle was found, together with the increased biosynthesis of fatty acids and decreased membrane proton motive force, in a ceftazidime-resistant *Vibrio alginolyticus* [27]. Colistin resistance in *V. alginolyticus* was also associated with the reduction in the P cycle. Metabolites in the P cycle reprogramed the colistin-resistant metabolome to a colistin-sensitive metabolome, resulting in increased gene expression, enzyme activity or protein abundance of the P cycle and sodium-translocating nicotinamide adenine dinucleotide-ubiquinone oxidoreductase [28]. In addition, the depressed P cycle contributes to the acquisition of ampicillin resistance in *Edwardsiella piscicida*, with the features of lower membrane potential and ATP [29]. To assay the fluctuation in the P cycle in Xoo-ZSM, the expression levels of genes involved in the P cycle were tested by qRT-PCR. As a result, four genes encoding SDH and two genes encoding KGDH declined in Xoo-ZSM. Furthermore, the activities of the SDH and KGDH were both reduced in Xoo-ZSM. The present results demonstrate that the depression of the P cycle was a feature of Xoo-ZSM. To our knowledge, this is the first report that reveals that the P cycle is associated with ZSM resistance development in the Xoo strain.

Finally, alanine was found as a crucial metabolite that significantly promotes the P cycle and reverses the ZSM resistance in Xoo. With the supplementary alanine in the culture broth, the P cycle in the ZSM-resistant strain was elevated and the bacterial survival percentages were decreased with the addition of exogenous alanine under ZSM stress. The effect of alanine on bacterial antibiotic resistance was consistent with the findings in several other microbes. In *Vibrio alginolyticus*, exogenous L-alanine reverts the reduced Na^+^-NQR complex and promotes gentamicin-mediated killing [30]. In *Edwardsiella tarda EIB202*, alanine metabolism connects to riboflavin metabolism, which provides the source for reactive oxygen species (ROS) production, and in turn, potentiates the killing of kanamycin-resistant bacteria [31]. Therefore, the function of alanine in enhancing the antibiotic-mediated killing in Xoo is similar to that found in the previous study. However, the specific mechanism of how the alanine functions in Xoo should be explored by more experimental verification. In addition, the mechanism of antibiotics resistance development may be infected by multi-factors, so more candidates able to reverse the ZSM resistance in Xoo should be discovered by further exploration in the future. Therefore, our works verified the positive role of alanine in reversing the antibiotic resistance and proposed a novel approach, that alanine could be applied as a candidate enhancer for ZSM to improve the antibiotic-mediated killing efficiency in Xoo. These results support the conclusion that an antibiotic-resistant metabolome can be reprogrammed to an antibiotic-sensitive metabolome by using crucial biomarkers to promote antibiotic-mediated killing efficacy [32,33,34].

To sum up, our findings highlight that the variation in metabolism is a crucial reason for the development of ZSM resistance in Xoo, while the depression of the P cycle can be drawn as a metabolic feature in the ZSM-resistant Xoo strain. Moreover, exogenous alanine reverses the depression of the P cycle, and enhances the efficiency of antibiotic-mediated killing. Our works not only reveal/partially reveal the mechanism of ZSM resistance in Xoo but also provide a novel strategy for controlling the ZSM-resistant Xoo in the field of crop protection.

## 4. Materials and Methods

### 4.1. Bacterial Strains and Culture Manipulation

Two *Xanthomonas oryzae* strains were used in this study, which were Zhongshengmycin (ZSM)-sensitive bacteria (Xoo-S) and its ZSM-resistant strain (Xoo-ZSM). Xoo-S strain was in our lab store, while Xoo-ZSM was obtained by continuous culture of Xoo-S under the stress of ZSM with the concentration of half of the MIC for 34 generations. In the antibiotic bactericidal assays, the ZSM-resistant strain refers to a Xoo whose MIC is 16-fold higher compared with the Xoo-S. PSA medium or broth (peptone 10 g/L, sucrose 10 g/L, sodium glutamate 1 g/L, agar 2% for solid medium, pH 7.0) were used for bacterial culture.

### 4.2. Determination of Minimum Inhibitory Concentration (MIC)

After being cultured on PSA medium for 3 days initially, Xoo-S and Xoo-ZSM were transferred to 5 mL PSA broth and incubated at 30 °C, 200 rpm for 24 h for seed culture. After that, 1% (*v*/*v*) products of the two strains were transferred into the 2 mL PSA fermentation broth with the addition of ZSM at gradient concentrations, followed by an 18 h shaken culture at 30 °C and 600 rpm. The OD_600_ value was observed by the MultiskanSky microplate instrument, and the MIC of ZSM to the strain was determined.

### 4.3. Measurement of Growth Curve

The 1% (*v*/*v*) products of the seed culture were transferred to 50 mL PSA broth, followed by the shaken culture at 30 °C, 200 rpm. The biomass was valued as the OD_600_ readings every 4 h. Each trial contained at least three biological repeats.

### 4.4. Sample Preparation of Metabonomics Based on GC-MS

Strains were cultured as mentioned above. After 32 h fermentation, the cultures were centrifuged at 4 °C, 12,000 rpm for 5 min. The bacterial cells were collected and washed with 0.9% normal saline 3 times. After washing, the bacterial solution OD_600_ was adjusted to 1.0 with 10 mL physiological saline. A total of 20 mL of frozen methanol (placed in the refrigerator at −20 °C for more than 24 h in advance) was added immediately to quench cells at 4 °C for 1 h to end cell metabolism. After that, the bacteria were collected and stored at −20 °C. Five biological replicates and two technical replicas for each strain were performed in this study to eliminate the background errors and the instrumental errors among the individual samples.

The samples for GC-MS analysis were prepared following the methods mentioned before [18]. Briefly, the resuscitated cells were transferred to the QSP microtubule of 1.5 mL and 0.1 mg/mL ribose was added as the internal standard. The cells were extracted for 6 min with 60% intensity sound waves, followed by centrifugation for 10 min at 4 °C and 14,200× *g*. The methanol was evaporated in a 37 °C vacuum centrifuge dryer. The dried samples were then mixed with 80 μL methoxide pyridine hydrochloride purchased from Sigma-Aldrich (20 mg/mL). After ultrasonic treatment, the reaction was carried out at 37 °C for 3 h, then 80 μL of N-methyl-N-trimethylsilyl trifluoroacetamide (MSTFA, SIGMA) was added followed by a 30 min reaction at 37 °C to obtain acidic protons. Finally, the samples were centrifuged for 5 min at 4 °C at 12,000× *g* and used for treatment on GC-MS. GC-MS data were detected by thermal science tracer DSQII.

### 4.5. Data Deposition and Statistical Analysis

The data deposition and statistic analysis were performed following the methods in the predecessors [35,36]. The total abundance of all metabolites in the sample was used as their relative abundance to measure the abundance of metabolites for further analysis. The compounds were identified according to the matching data of the National Institute of Standards and Technology (NIST) library and NIST MS search 2.0 program, and the mass spectrometry was analyzed by X Calibur software (ThermoFisher, version 2.1, Waltham, MA, USA). The data were normalized by total amount of correction and standardized data including metabolites, retention time, and peak area, which was convenient for further metabolomic analysis. The metabolites were analyzed by R software (R × 643.6.1). Principal component analysis (PCA) and S-Plot analysis were carried out with SIMCA-P+12.0 software (Version12; Umetrics, Umea, Sweden), and then each metabolite was graded and the average value and standard deviation were calculated to obtain the Z-SCORE analysis. SPSS statistics 19 software and Graph Prism 7 Project were used to analyze the significant differences in the standardized data, and the metabolites with differences were screened out (*p* < 0.05). The metabolic pathway was enriched by MetaboAnalyst4.0.

### 4.6. Antibiotic Bactericidal Experiments

The ZSM-resistant strain was cultured as described above. The well-cultured bacterial cells were collected at 8000 rpm for 5 min, washed three times with 30 mL sterile saline, and then suspended in PSA broth for 18 h culture. During this culture phase, ZSM with/without alanine was added into the PSA broth to determine the influence of alanine on ZSM killing efficiency. The alanine dosages were set from 0 to 40 mM. After incubation, 100 μL of samples were serially diluted and plated (5 μL aliquots) onto PSA agar plates. The plates were cultured at 30 °C for 60–70 h depending on bacteria species. To determine colony-forming units (CFU) per mL, only those dilutions yielding 20–200 colonies were enumerated. Percent survival was determined by dividing the CFU obtained from a treated sample by the CFU obtained from control.

### 4.7. Real-Time Quantitative PCR

The strains were cultured according to the method described above, and the expression level of related genes in the pyruvate cycle was detected by quantitative real-time PCR (qRT-PCR). Briefly, the total RNA (Dobiothec, Fuzhou, China) of *Xanthomonas oryzae* was extracted according to the Biospin Total RNA Extraction Kit step. The concentration and purity of total RNA of *Xanthomonas oryzae* were measured by μDrop plate, and the cDNA of *X. oryzae* was obtained by One-Step gDNA Removal and cDNA Synthesis SuperMix Kit (TransGen Biotech, Beijing, China). qRT-PCR was carried out on a 96-well plate containing 20 μL of liquid per well, including 10 μL 2× Perfect start Green qPCR SuperMix, 0.4 μL 50× Passive Reference Dye optional, 6.8 μL nuclease-free water, 2 μL cDNA template, and 0.4 μL per pair of specific primers. The detailed information of the primers is listed in Table S1. To analyze the relative expression level of the target gene, we converted the data to a percentage relative to the control group, and the data were calculated following the method mentioned before [16,37]. At least three biological replicates were carried out.

### 4.8. Measurement of the Activity of Enzymes in the P Cycle

The cell strains and the appropriate amount of extract (the ratio of 5 million cells/1 mL extract) were mixed and crushed by ultrasonic oscillation. The cells were broken with 60% intensity sound waves at 4 °C for 6 min, followed by centrifugation at 12,000× *g* for 10 min, and the supernatant was transferred to a new centrifuge tube. The BCA protein quantitative/concentration determination kit (Meilun, Dalian, China) was used to obtain the protein standard curve so that the concentration of the protein sample could be detected. The activities of KGDH and SDH were determined by KGDH and SDH detection kits (Solabio, Beijing, China), respectively.

## Data Availability

Not applicable.

## Supplementary Materials

The following are available online at https://www.mdpi.com/article/10.3390/antibiotics11020245/s1, Figure S1: The total metabolites detected in Xoo-S and Xoo-ZSM, Figure S2: Pattern recognition and identification of crucial metabolites, Figure S3: The relative abundances of the 9 candidate crucial metabolites, Table S1. the primers used in this study for qRT-PCR.
