# Supplementary material for: Exogenous Alanine Reverses the Bacterial Resistance to Zhongshengmycin with the Promotion of the P Cycle in Xanthomonas oryzae
Source: Antibiotics (Basel). 2022 Feb 14;11(2):245. doi: 10.3390/antibiotics11020245 (PMC8868265; doi:10.3390/antibiotics11020245)
Supplement: Supplementary file 1 [file antibiotics-11-00245-s001.zip › antibiotics-1559484-supplementary.pdf]

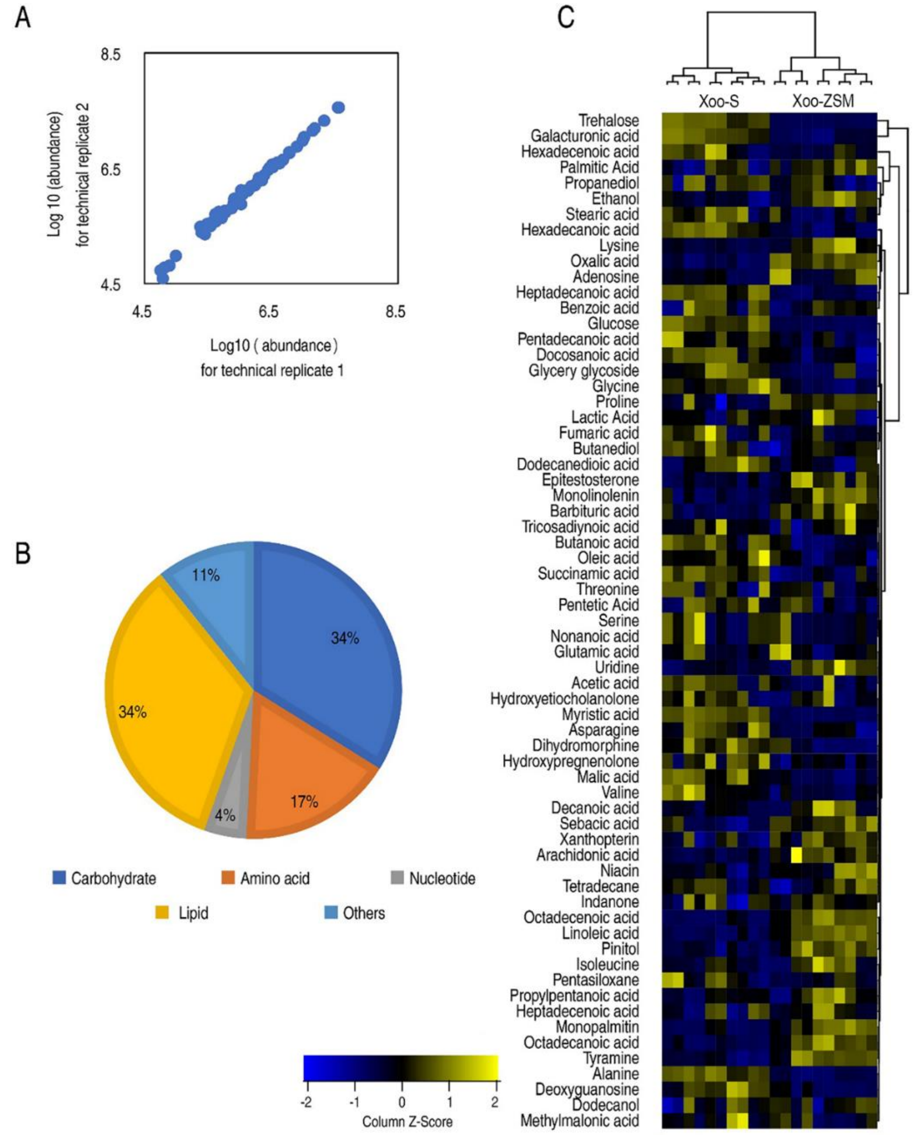

Figure S1. The total metabolites detected in Xoo-S and Xoo-ZSM. (A) Reproducibility of metabolic profiling platform used in the discovery phase. Correlation coefficient were higher than 0.995 between technical replicates. (B) Percentage of the total metabolites in every category. (C) Heat map of differential abundance metabolites. Yellow and blue indicate the increase and decrease of the metabolites scaled to mean and SD of row metabolite level, respectively (see color scale).

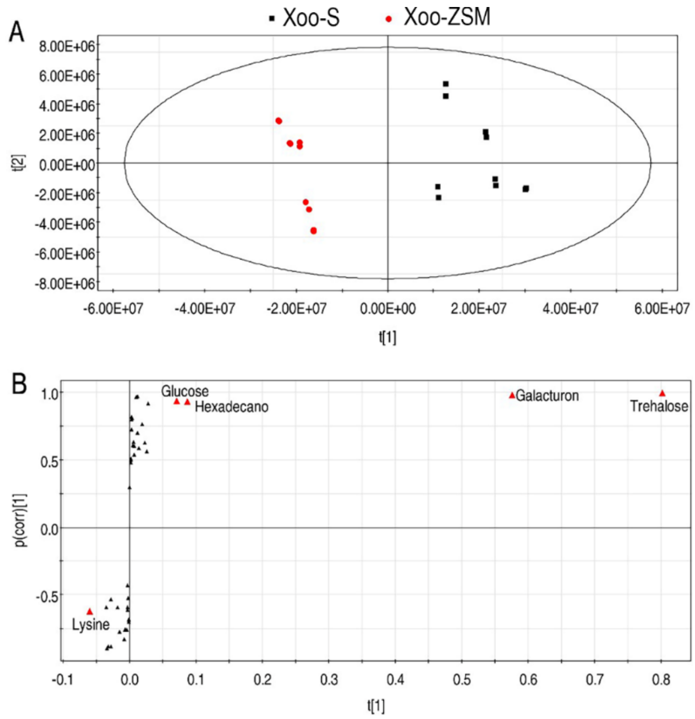

Figure S2. Pattern recognition and identification of crucial metabolites. (A) Score plot of the OPLS-DA model from all detected metabolites. (B) OPLS-DA loadings plot for metabolites.

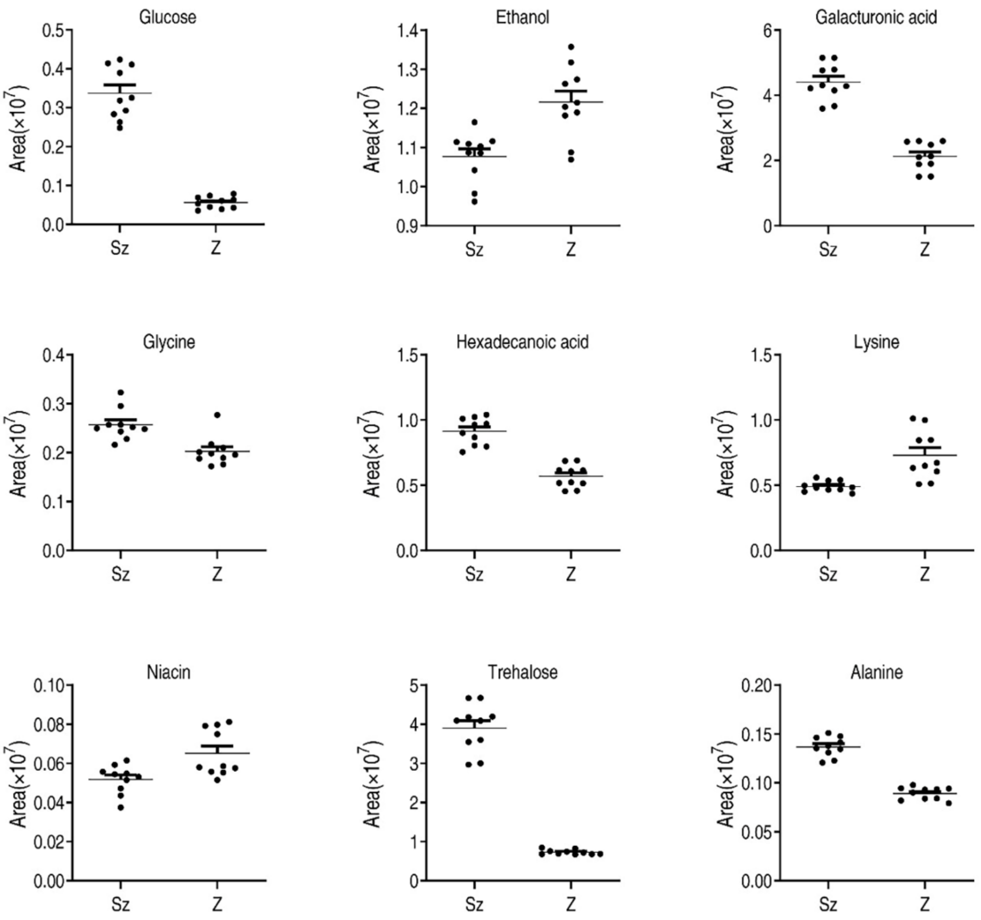

Figure S3. The relative abundances of the 9 candidate crucial metabolites.

Table S1. the primers used in this study for qRT-PCR.

Primers	Sequences (5′-3′)	Annotation
SDH-hmap-F/R	GCAGGTGGTGCTGGAAGATTACG/ ACGGCAAACACGCTGACGATG	succinate dehydrogenase
SDH-fs-F/R	GCTGCTGGATCTGGTGGTGTTC/ AAGGTCTTGTGCGGCTGATTGG	
SDH-b556-F/R	CGTTCGTCGGCATGGGTTGG/ TACGGCACCTCCAGACAGCAG	
SDH-iron-F/R	TGAACATCGACGGCACCAATACG/ GCAGCGGATAGATCGGCACTTC	
KGDH-E1-F/R	GCACGACCAGAAGACCGACAAC/ CCATCACCGCTTCTTCGCTGAG	α-ketoglutarate dehydrogenase
KGDH-E2-F/R	GCCAGTCGTTCGCCGATGTC/ AAGGTGCCGCCATTGGTGATG	
